# Understanding Pre-Service Teachers’ Perceptions of the Ideal Learning Environment for Mathematical Activities Through Drawings

**DOI:** 10.3390/bs15040517

**Published:** 2025-04-13

**Authors:** Tuğba Yulet Yılmaz, Mustafa Gök

**Affiliations:** Department of Mathematics and Science Education, Faculty of Education, Van Yuzuncu Yil University, 65080 Van, Türkiye; mustafagok@yyu.edu.tr

**Keywords:** mathematical activities, learning environment, pre-service teachers, drawings

## Abstract

Mathematical activities, which have the potential to engage students cognitively, are an essential component of modern educational approaches. The design of learning environments that facilitate the full realization of the potential of mathematical activities is as crucial as the activities themselves. Well-designed physical, social, and emotional learning environments significantly contribute to students’ holistic development. Pre-service teachers’ perceptions regarding the ideal learning environment for mathematical activities can shape the quality of learning environments they will create professionally. This study explores the perceptions of pre-service primary school teachers and pre-service elementary mathematics teachers regarding the ideal learning environment for mathematical activities through their drawings and related reflections. This study employs a qualitative case study design, and data were collected through freehand drawings depicting ideal learning environments and semi-structured interviews with pre-service teachers. The analysis reveals five prominent components of the ideal learning environment for mathematical activities: purpose, instructional methods and techniques, teacher–student roles, seating arrangements, and interrelations among these components. The findings indicate differences in perceptions between pre-service primary school teachers and pre-service elementary mathematics teachers. Additionally, the results highlight that these components are interrelated within the learning environment, with the purpose component serving as a guiding factor for the others.

## 1. Introduction

The process of teaching and learning mathematics is influenced by various factors. These factors encompass a country’s educational philosophy and students’ prior knowledge, misconceptions, learning difficulties, and socioeconomic backgrounds. In parallel with these variables, teachers’ pedagogical decisions and the strategic steps they take are also shaped by multiple influences. The selection of mathematical activities and their implementation within an interactive learning environment, where a positive classroom climate is fostered, are direct outcomes of these pedagogical decisions.

Mathematical activities are social practices jointly carried out by teachers and students, allowing learners to engage actively with mathematical content (Doyle, 1983; Johnson et al., 2017). Within a mathematics classroom, mathematical activities play a crucial role in elevating the instructional standards (Barber, 2018). This significance stems from their potential to encourage students to engage in reasoning, problem-solving, making connections, constructing arguments, and participating in discussions (Doyle, 1988; Lee et al., 2013; Mason et al., 2009; Smith & Stein, 1998; Sullivan, 2011). In this context, mathematical activities not only provide students with the opportunity to take responsibility for their learning but also place upon teachers the responsibility of selecting and implementing high-quality activities that support students’ mathematical thinking (Rogers & Freiberg, 1994). The National Council of Teachers of Mathematics (NCTM, 2014) defines the selection and implementation of mathematical activities as a fundamental element of effective mathematics teaching and directly links this process to teacher competencies.

The learning environment in which they are implemented is as crucial as the selection of mathematical activities. Research has consistently demonstrated a strong relationship between learning environments and learning outcomes (Woolner et al., 2007). Teachers play a critical role in designing learning environments that optimize mathematics learning, determining the essential elements to be included, and ensuring that these environments support student engagement and understanding. It is evident that teachers’ knowledge and perceptions of mathematical activities directly influence every stage of the selection and implementation processes (Adleff et al., 2023).

As integral components of the learning environment, teachers play a key role in fostering a positive classroom climate that enhances students’ motivation and sense of effectiveness (Velayutham et al., 2013). Teachers’ perceptions of innovative and constructivist learning environments significantly impact various pedagogical decisions, including the selection of mathematical activities, their implementation processes, the roles of students and teachers, and the determination of instructional materials. In addition to in-service teachers, pre-service teachers’ perceptions also have the potential to shape future mathematics teaching practices. At this point, the role of pre-service teachers’ perceptions in creating learning environments that facilitate the effective implementation of mathematical activities, which have the potential to cognitively engage students, is of critical importance.

Various methodological approaches and frameworks can be employed to explore teachers’ perceptions of learning environments (Anagun, 2018; Tunçsiper & Mutlu, 2020; Uygun & Duman, 2022). These approaches provide insights into how teachers conceptualize and interact with their instructional settings (Tunçsiper & Mutlu, 2020). Among these, drawings offer a unique visual representation of teachers’ mental models and perceptions, allowing for a deeper understanding of their views on learning environments (Bland, 2018; Hatisaru et al., 2023; Kuzle, 2023). It is assumed that the drawings of an ideal learning environment created by pre-service teachers can shape the learning environments they will establish professionally. In this regard, physical, educational, and socio-emotional elements are closely related to how pre-service teachers perceive and experience a learning environment. Understanding the ideal learning environments envisioned by future teachers requires further research, particularly in the context of pre-service primary school teachers and pre-service elementary mathematics teachers who will integrate mathematical activities into their classrooms.

Given the significance and necessity of this issue, this study aims to explore pre-service teachers’ perceptions of the ideal learning environment for mathematical activities through their drawings and related reflections.

### 1.1. Understanding the Components of an Ideal Learning Environment

Learning environments are complex structures that integrate the physical, social, psychological, intellectual, pedagogical, and cultural dimensions in which students learn and where learning takes place (Fraser, 2007; Kuzle, 2023). These environments serve as a critical source of motivation for students’ holistic development in school and represent a fundamental factor influencing learning strategies and engagement levels (Cayubit, 2022). In all subjects, including mathematics, the learning environment plays a decisive role in increasing students’ interest and motivation toward the lesson (Gilbert et al., 2014).

The interplay of student–teacher–physical space shapes an ideal learning environment and should possess characteristics that support students, promote equity, facilitate learning, and maximize educational outcomes (Zedan, 2010). Getzels (1974) classified learning environments into four categories: traditional, active, open, and social. In this classification, teacher-centered environments are defined as traditional, student-centered environments as active, out-of-school learning environments as open, and interactive settings as social. However, considering the advancements in science and technology alongside educational reforms, it is evident that this classification is insufficient in capturing the nature of contemporary learning environments. Various new approaches, such as constructivist, online, hybrid, experiential, innovative, and informal learning environments, have gained popularity in recent years.

According to García-Rodríguez et al. (2024), learning environments not only contribute to students’ socialization processes but also include physical elements, teachers’ instructional methods, students’ learning strategies, and various interaction and communication components. These environments influence key factors such as attitudes, values, skills, and motivation. Accordingly, studies have linked high-quality learning environments to positive outcomes, including academic achievement, engagement, self-concept, skill development, satisfaction with learning experiences, the quality of peer communication, and emotional well-being (Guo et al., 2021; S. Lin et al., 2018; McMinn et al., 2021; Ozkan Bekiroglu et al., 2022; Rusticus et al., 2023; Vermeulen & Schmidt, 2008).

Different researchers emphasize various components of effective learning environments, often asserting that the design of these environments is shaped by the intended learning objectives. According to Anderson and Krathwohl (2001), clearly defined goals that align with both content and process facilitate a focus on learning, define the roles of teachers and students, and enhance achievement. In this regard, learning objectives not only guide the learning process but also influence the choice of instructional methods and the design of learning environments. Similarly, Hattie (2009) underscores the strong impact of learning strategies, student–teacher relationships, explicitly stated learning goals, and well-defined success criteria on students’ conceptual understanding and academic performance.

An ideal learning environment holistically addresses students’ psychological, social, cultural, and physical needs, fostering interaction and motivation (Zadina, 2023; Rusticus et al., 2020). By supporting students’ cognitive, neurological, and emotional development, this environment enhances active engagement in the learning process.

To understand the structure of learning environments, Schönrock-Adema et al. (2012) recommend Moos’ framework of human environments (Moos, 1973, 1991). This framework examines all environments through three analytical dimensions: personal development/goal orientation, relationships, and system maintenance/change. The personal development dimension refers to the potential for personal growth within the environment, including its emotional climate and its role in fostering self-esteem. The relationship dimension pertains to the nature and quality of social interactions, reflecting the level of engagement, cooperation, and mutual support among individuals in the environment. The system maintenance/change dimension encompasses the degree of structure, clarity, and openness to change within the environment, including its physical characteristics.

Building on Moos’ framework, Rusticus et al. (2023) applied these dimensions to the context of university learning environments. The personal development dimension encompasses factors that enhance students’ motivation both within and beyond the classroom, including engagement in learning and work–life balance. The relationship dimension highlights academic and social support mechanisms, such as faculty guidance, peer interaction, and collaborative learning. Finally, the institutional structure corresponds to Moos’ system maintenance and change dimension, encompassing physical spaces, institutional expectations, and the overall cultural climate, addressing aspects such as the significance of small class sizes and the sense of community.

In the present study, drawings were utilized to explore how pre-service teachers conceptualize an ideal learning environment in which instructional activities are implemented.

### 1.2. The Role of Drawings in Educational Research

Drawings serve as a unique tool for uncovering the perceptions of learning environments. They not only provide insights into the content being represented but also reveal aspects of the individual’s personality, knowledge level, skills, beliefs, and attitudes (Damianov, 2022). Weber and Mitchell (2002) emphasize that drawings can expose thoughts that are difficult to articulate, subconscious elements, and abstract ideas that may be challenging to grasp. Therefore, drawings are particularly valuable for individuals who struggle to express their thoughts verbally or in writing (Briell et al., 2010).

In educational research, drawings are widely used to assess student and teacher perceptions. For instance, the Draw-a-STEM Learning Environment (D-STEM) tool, designed to examine university academics’ perceptions of STEM education, has revealed a limited understanding of interdisciplinary learning among participants (Hatisaru et al., 2023). Similarly, elementary students’ drawings of their geometry classrooms have indicated a teacher-centered environment with limited interaction, highlighting the role of drawings in assessing the psychosocial dimensions of classroom climate (Kuzle, 2023). Findings derived from pre-service teachers’ drawings have demonstrated changes in their perceptions of past, present, and idealized future classroom experiences (Albert, 2012).

For children and adults, drawings provide a valuable approach to accessing individuals’ imagination and understanding their perspectives. Vygotsky (2004) argues that drawings serve as one of the most accessible means for children to express situations that concern them. Children can convey their thoughts and perceptions more clearly through drawings, making them a crucial tool for understanding emotions and ideas (Chang, 2012; Hope, 2008). McHatton et al. (2014) further assert that students’ drawings of learning environments offer valuable insights that may contradict teachers’ perceptions, playing a critical role in identifying factors that either support or hinder learning.

Overall, drawings represent a powerful and creative approach for gaining in-depth insights into perceptions of learning environments and enhancing educational practices.

### 1.3. Theoretical Framework

Teacher perceptions play a fundamental role in shaping the physical, social, and pedagogical characteristics of learning environments. These perceptions not only influence the design of classroom settings but also determine their impact on learning. Woolner and Hall (2010) emphasize that noise levels in physical learning environments can negatively affect students’ learning experiences. Similarly, Byers et al. (2018) explore the influence of physical settings on pedagogical transformation, highlighting the potential of innovative learning environments to reshape teachers’ instructional approaches. Tunçsiper and Mutlu (2020) argue that teacher perceptions tend to focus primarily on physical aspects, directly affecting classroom design and instructional practices. In this regard, Dalinger et al. (2024) demonstrate that teachers’ perceptions of classroom conditions influence job satisfaction and professional competence, with positive classroom climates enhancing teacher motivation. Likewise, Poyato-Nunez et al. (2024) emphasize that well-designed physical learning environments support innovative teaching approaches, thereby enriching classroom practices.

Beyond the physical environment, teacher perceptions also influence the social and pedagogical dimensions of learning spaces. Stăncescu et al. (2016) emphasize that student-centered approaches, which take into account prior knowledge, contribute to the development of learning environments that promote collaboration and communication skills. Integrating technology into educational settings plays a crucial role in this process. For example, Ozkan Bekiroglu et al. (2022) argue that flexible, technology-enhanced learning environments promote cognitive and emotional engagement, strengthening teacher–student interactions. In this context, teachers’ skills and competencies significantly shape how they manage technological integration and define their roles within learning environments.

Moos (1974) provides a comprehensive framework for analyzing learning environments by identifying three key dimensions that structure human environments: relationship dimensions, personal development or goal orientation dimensions, and system maintenance and change dimensions. Relationship dimensions reflect the nature and intensity of social interactions, personal development dimensions emphasize opportunities for growth and self-esteem, and system maintenance/change dimensions highlight structure, organization, and adaptability within an environment (Insel & Moos, 1974). In the context of this study, which examines pre-service teachers’ perceptions of ideal learning environments, Moos’ model serves as a foundational framework. Understanding how pre-service teachers conceptualize teaching methods, student roles, and classroom structures aligns with the three dimensions of Moos’ model. For instance, their preferences for collaborative activities reflect the relationship dimension, their emphasis on student-centered learning relates to personal development, and their views on classroom organization correspond to system maintenance/change. Moreover, findings from previous research indicate that a lack of community within a learning environment can negatively impact identity and engagement (Rusticus et al., 2023), reinforcing the need for environments that foster meaningful interactions and a sense of belonging.

Stylianidou et al. (2020) assert that the incorporation of augmented reality and alternate reality games into education, in alignment with inclusive education principles, offers accessible and transformative learning experiences. Similarly, Hasanein and Sobaih (2023) highlight that artificial intelligence tools such as ChatGPT serve as versatile resources in dynamic learning environments. The effective utilization of such innovative technologies is closely linked to teachers’ pedagogical competencies. For instance, Dag et al. (2019) report that teachers who adopt active learning models exhibit more positive perceptions in areas such as cognitive awareness, collaboration, and engagement. Similarly, Zhang and Morselli (2016) emphasize that teachers’ subject matter knowledge and pedagogical skills shape technological integration processes and overall instructional effectiveness.

Research focusing on the social and pedagogical dimensions of online and interactive learning environments underscores the importance of fostering student engagement in instructional processes. Lasekan et al. (2024) argue that sustainable online learning depends on high-quality content delivery and interactive pedagogical practices. This aligns with Anagun’s (2018) assertion that teachers equipped with problem-solving, critical thinking, and collaboration skills create more effective learning environments. Similarly, Almusharraf (2024) emphasizes that enhancing the usability of learning management systems significantly contributes to increased student satisfaction and engagement. Niemi (2021) further emphasizes the importance of socially responsive learning environments that prioritize students’ needs in shaping learning experiences.

Teachers’ competencies play a pivotal role in effectively integrating the social, pedagogical, and technological dimensions of learning environments. McMinn et al. (2021) report that pre-service teachers’ self-efficacy in mathematics teaching and their beliefs about mathematics directly impact student engagement and instructional processes. In this regard, teachers’ positive perceptions, supported by strong pedagogical skills, contribute to the success of innovative learning environments.

These studies demonstrate that teachers’ perceptions shape learning environments and influence students who share these spaces. Magen-Nagar and Steinberger (2017) found that students’ perceptions of innovative learning environments align with the constructivist approach, emphasizing student engagement, research opportunities, collaboration, teacher support, and technology use. Notably, alignment between teacher and student perceptions of learning environments contributes to a positive classroom climate. However, Könings et al. (2014) report that while teachers ideally envision student-centered and supportive learning environments, their perceptions often do not align with those of students. According to researchers, this misalignment may lead to motivational and academic challenges.

Visual representations, particularly drawings, are a powerful tool for uncovering individuals’ perceptions. Drawings not only reflect an individual’s knowledge level but also reveal their beliefs, attitudes, and emotions (Damianov, 2022). Kuzle (2023) examined elementary school students’ psychosocial perceptions of mathematics learning environments through drawings. Farmer et al. (2018) used this method to investigate students’ emotional experiences and social interactions within classrooms. Numerous studies based on children’s drawings suggest that drawings reflect children’s beliefs (Anning, 2004), mathematical perceptions (Matney et al., 2023), and attitudes toward mathematics (Quane et al., 2023). In addition to revealing students’ perceptions of their current learning environments, drawings can also illustrate their mental representations of ideal learning spaces. For instance, Bland and Sharma-Brymer (2012) analyzed Australian children’s drawings of ideal learning environments, examining school features and learning space preferences. Their findings suggest that children’s ideas can inform the creation of spaces that foster more engaging pedagogical interactions and student-centered instructional styles.

Beyond children’s drawings, adult-generated drawings also provide valuable insights (Barrantes-Elizondo, 2019). Supporting this notion, Satyam et al. (2022) argue that drawings can facilitate meaningful discussions by offering pre-service teachers experiential learning opportunities. Kuvac and Koc (2023) used drawings to explore how STEM education shapes pre-service teachers’ perceptions of engineering. Wescoatt (2016) examined pre-service teachers’ mental models while engaging with mathematics through drawings. Y. C. Lin (2022) emphasized the role of drawings in understanding pre-service teachers’ beliefs, pedagogical knowledge, and emotions related to mathematics. Eroğlu and Arslan (2024) employed drawings to investigate pre-service teachers’ beliefs about ideal mathematics learning environments.

Recent studies have demonstrated that drawings can serve as a powerful methodological tool for uncovering PSTs’ prior knowledge, attitudes, and evolving professional identities (Keren-Kolb & Fishman, 2006; Akerson, 2016; Katz et al., 2011). Keren-Kolb and Fishman (2006) emphasized that teacher education programs face challenges in preparing PSTs for technology integration, partly due to the deeply ingrained prior beliefs they bring into their training. Their study utilized drawings and interviews to illuminate these beliefs, offering a qualitative alternative to traditional survey-based approaches. Similarly, Akerson (2016) investigated PSTs’ perceptions of mathematics through pre- and post-field experience drawings. The findings highlighted shifts in emotional responses, learning environments, and engagement with mathematics, suggesting that structured field experiences can positively influence PSTs’ perspectives. In science education, Katz et al. (2011) explored how participation in informal afterschool science internships influenced PSTs’ professional identities. Their drawings and narratives revealed a transition from teacher-centered to student-centered instructional approaches, emphasizing inquiry-based and collaborative learning.

These studies collectively demonstrate that drawings serve as a valuable tool for understanding individuals’ perceptions of their current learning environments and their idealized visions of such spaces. Therefore, drawings offer a creative and effective method for enhancing learning environments.

### 1.4. Current Study

In the literature, the critical importance of mathematical activities in mathematics education has been emphasized (Barber, 2018; Doyle, 1983; Johnson et al., 2017), and the multifaceted effects of learning environments on students have been explored (García-Rodríguez et al., 2024; Ozkan Bekiroglu et al., 2022; Rusticus et al., 2023). In particular, teacher perceptions have been found to shape instructional processes and the design of learning environments (Adleff et al., 2023; Zhang & Morselli, 2016). Moreover, drawings have been recognized as a powerful and creative method for examining individuals’ perceptions (Bland, 2018; Satyam et al., 2022).

This study explores the perceptions of pre-service primary school teachers and pre-service elementary mathematics teachers regarding the ideal learning environment for mathematical activities through their drawings and related reflections. In this context, pre-service teachers were asked to depict their envisioned ideal learning and teaching environment through drawings and to support their representations with explanations and reflections. Drawings are an effective tool for understanding the individual components of an ideal learning environment envisioned by participants and for systematically evaluating these components. This study seeks to make a significant contribution to understanding pre-service teachers’ perceptions of mathematical activities and learning environments through visual representations.

Accordingly, this study addresses the following research questions:

- What are the perceptions of pre-service primary school teachers regarding an ideal learning environment that incorporates mathematical activities?
- What are the perceptions of pre-service elementary mathematics teachers regarding an ideal learning environment that incorporates mathematical activities?
- How do the perceptions of pre-service primary school teachers and pre-service elementary mathematics teachers differ concerning an ideal learning environment that incorporates mathematical activities?

## 2. Materials and Methods

In this study, a qualitative research method, a case study, has been adopted. According to Merriam (2018), the case study is a research method providing an in-depth exploration of an event, phenomenon, or process within a specific context. This approach analyzes and interprets the situation being studied in great detail. Critical qualitative research in mathematics education emphasizes relationships and non-traditional communication methods, contributing to creating a more inclusive and justice-oriented research environment (Westby, 2024). In this context, the research phenomenon has been addressed by synthesizing interpretive and critical orientations (Schwandt & Gates, 2018). The case examined in this study is pre-service teachers’ perceptions of an ideal mathematics learning environment in which mathematical activities are utilized. One of the reasons researchers chose this design is that the case represents a current issue addressed within a real-world context. Another reason is that the researchers aim to conduct an in-depth analysis of this case to present a holistic perspective on the subject.

### 2.1. Participants

The study participants consist of pre-service primary school teachers and pre-service elementary mathematics teachers enrolled at a public university in Türkiye. The pre-service elementary mathematics teachers follow a more intensive curriculum focused on mathematics teaching, including courses on the fundamentals of mathematics, geometry and measurement instruction, probability, and statistics. In contrast, the curriculum for pre-service primary school teachers includes mathematics teaching and courses across a broader range of disciplines (Council of Higher Education [CoHE], 2018).

The participants were selected using a purposive sampling method based on specific criteria that align with the research problem (Palinkas et al., 2015). The selection criteria were as follows:

- The successful completion of core undergraduate courses related to mathematics education;
- The necessity to design and implement mathematical activities;
- Voluntary participation in this study.

These selection criteria ensured that the participants’ knowledge and experience in mathematical activities were suitable for the aims of this study. Detailed demographic information about the participants is presented in Table 1.

When examining demographic characteristics across groups, most participants fall within the 22–24 age range. The gender distribution is skewed toward female students in both groups. Additionally, while pre-service elementary mathematics teachers are generally in their fourth year, pre-service primary school teachers are in their third year. Participation in extracurricular education programs is low in both groups. These programs vary and include topics such as multi-grade classrooms, teaching practice, positive error climate, mind and intelligence games, and drama. Nevertheless, although the mathematics curricula in Türkiye emphasize the constructivist approach in schools, both pre-service primary school teachers and pre-service elementary mathematics teachers generally learn about the constructivist approach theoretically in their undergraduate courses. There are several reasons why undergraduate students’ courses are conducted using traditional methods. Factors such as overcrowded classrooms, desks and tables being fixed to the floor, the presence of only a projector in most classrooms, and the lack of an internet connection are among these reasons. For all these reasons, it is a fact that both pre-service primary school teachers and pre-service elementary mathematics teachers encounter fewer examples of constructivist learning approach applications in their undergraduate courses.

### 2.2. Data Collection Instruments and Procedure

In this study, data were collected through documents obtained from drawings and semi-structured interviews conducted with each pre-service teacher in the middle of the fall semester course process of the 2024–2025 academic year. A free drawing activity was implemented to determine pre-service teachers’ perceptions of and visions for the ideal learning environment in which mathematical activities would be conducted. Notably, free drawing is recognized as a standalone method within a range of visual data collection techniques (Bland, 2018). Albert (2012) stated that drawings can serve as a tool for examining the underlying thoughts behind pre-service teachers’ pedagogical practices. Drawings are acknowledged as a suitable data collection tool, similar to other qualitative research instruments (Gulek, 1999; Russell, 1999), and explanations accompanying drawings have been noted to yield more valid and reliable results (Armstrong, 1995). During this process, participants were allowed to freely create their drawings and were encouraged to provide brief explanations about them (Utley et al., 2020). Thus, drawings, which allow pre-service teachers to express their thoughts, constitute a significant data source (Eroğlu & Arslan, 2024; Satyam et al., 2022).

In the present study, the researchers asked participants to visually depict their ideal learning environment as they envisioned it and informed them that they could add speech bubbles to their drawings. The pre-service teachers completed their drawings in approximately 40 min. The data obtained from these drawings were used to analyze in detail the participants’ perceptions of the ideal learning environment. Additionally, these drawings were considered a tool for understanding various aspects of the participants’ perceptions regarding the use of mathematical activities. Following the drawing activity, the researchers conducted semi-structured interviews with the participants, during which they were asked to explain their drawings. Initially, all participants were asked the question, “Can you describe your drawing of an ideal learning environment where mathematical activities are used?” during the interviews. If the participant’s drawing and explanation were insufficient, the researchers asked probing questions to obtain further clarification. The interview questions asked to the participants in the study are provided below:

- Can you describe your drawing of an ideal learning environment where mathematical activities are used?
  ○ Why did you design your drawing this way?
  ○ What are the students doing in your drawing?
  ○ What is the teacher doing in your drawing?
  ○ What is on the desks?
  ○ For what purpose did you use this material?
  ○ Why did you choose this seating arrangement?

The interviews aimed to concretize the participants’ pedagogical perceptions, facilitate the interpretation of visual data, and understand how they structured their teaching strategies. This approach also helped prevent the misinterpretation of the drawings beyond their intended meanings. During the data collection process, one of the researchers was the course instructor for the pre-service elementary mathematics teachers, while the other was responsible for instructing the pre-service classroom teachers. Accordingly, each researcher conducted interviews with the group of pre-service teachers they were teaching in order to maintain familiarity and ensure a comfortable interview environment. The interviews were conducted in the participants’ own classrooms after they completed their drawings. Only a voice recorder was used during the interviews. After collecting the papers with the participants’ drawings, the researchers individually called each participant for an interview and asked questions based on their drawings. The interviews lasted between 5 and 10 min.

Before implementation, the researchers obtained ethical approval for this study from the university’s Social and Human Sciences Ethics Committee. Additionally, the pre-service teachers were informed in detail about the study’s purpose, the use of the collected data, and confidentiality protocols. The researchers used code names instead of the participants’ real names (e.g., PPST P1: pre-service primary school teacher; PEMT P1: pre-service elementary mathematics teacher). The researchers also emphasized that participants had the right to withdraw from the study at any time, and those who agreed to participate signed an informed consent form.

### 2.3. Data Analysis

The data were analyzed using the two-cycle coding approach proposed by Saldana (2023). In the first cycle, the researchers independently conducted open coding by identifying concepts, patterns, themes, and categories from the raw data obtained from the drawings and the semi-structured interviews related to the drawings. This initial coding phase resulted in a general code list that captured the essence of the data. The development of this code list was informed by prior studies in the literature focusing on learning environment drawings (Eroğlu & Arslan, 2024; Farmer et al., 2018; Kuzle, 2023; Moos, 1973, 1991).

Following the first cycle, a second-cycle coding process was conducted to organize the codes into categorical, thematic, and conceptual structures (Saldana, 2023). In this stage, the researchers employed thematic analysis to establish a structured framework for understanding the data by identifying, analyzing, and reporting patterns. During the second-cycle coding, the researchers derived the themes “purpose, instructional methods and techniques, student role, teacher role, and seating arrangement” and categorized the codes under these themes. Saldana (2023) highlights that this two-cycle approach enhances the validity and reliability of qualitative findings, reduces individual bias, and increases confidence in the results by incorporating multiple perspectives. The researchers’ identified themes and sub-themes are presented in Table 2:

The themes and sub-themes identified in this study were structured within the framework of Moos’ three-dimensional model of human environments: personal development/goal direction, relationships, and system maintenance/change. This organization allows for a comprehensive analysis of the learning environment by categorizing the key aspects of teaching and learning processes. In developing these themes, prior studies such as Kuzle (2023), Eroğlu and Arslan (2024), Albert (2012), Lindstrom et al. (2021), and Getzels (1974) were utilized. While these studies provided fundamental insights, the thematic structure in this study also incorporates original contributions.

Within this framework, the purpose theme aligns with the personal development/goal direction dimension as it encompasses elements that foster student motivation, engagement, and conceptual understanding. The student role and teacher role themes, along with certain aspects of teaching methods and techniques, are categorized under the relationships dimension, emphasizing interactions, collaboration, and support systems in the learning environment. Meanwhile, the seating arrangement theme and the remaining aspects of teaching methods and techniques correspond to the system maintenance/change dimension, as they pertain to the structural and organizational characteristics of the learning environment.

**Figure 1:**
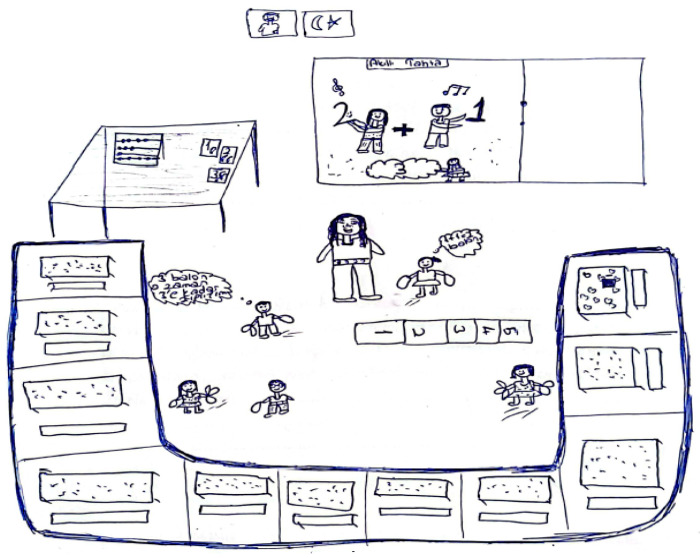
An example of a pre-service teacher’s drawing.

Figure 1 presents an example of a pre-service teacher’s drawing of an ideal learning environment for implementing mathematical activities, along with a sample coding of data obtained from the interviews.

- Theme: Purpose

The pre-service teacher drew an abacus and number–picture cards on the teacher’s desk. During the interview, the pre-service teacher stated that the abacus was drawn to help students concretize numbers and operations, while the number–picture cards were intended to relate numbers to daily life. The classroom was equipped with an interactive whiteboard, which included number-related visuals and an addition song. The pre-service teacher mentioned that this integration aimed to make the topic more engaging and enjoyable (Sub-Themes: Concrete Representation of Mathematical Concepts, Relating to Daily Life, Integrating Technology, Developing a Positive Attitude toward Mathematics).

- Theme: Teaching Methods and Techniques

A hopscotch game was drawn in the middle of the classroom. During the interview, the pre-service teacher explained that this game was designed to reinforce addition through bodily activities (sub-theme: Game-Based Learning).

- Theme: Student Role

All students actively participated in the game in the center of the classroom. Each student held a balloon, and one of them was observed saying, “Three balloons, so jump up to three.” The students were smiling, indicating that they were learning with enjoyment (sub-themes: Behavioral Engagement and Emotional Engagement).

- Theme: Teacher Role

The teacher was among the students, providing guidance. The pre-service teacher stated that the teacher acted as a facilitator, making the lesson enjoyable through games and music while offering emotional support (sub-themes: Facilitator and Emotional Support Provider).

- Theme: Seating Arrangement

The classroom was arranged in a U shape. The pre-service teacher explained that this arrangement was chosen to create an environment where students could comfortably engage in activities (sub-theme: U-Shaped Arrangement).

The emerging themes have been considered as different components of a learning environment, and the findings have been presented under five distinct components: “purpose component”, “teaching methods and techniques component”, “student roles component”, “teacher roles component”, and “seating arrangement component”.

### 2.4. Validity and Reliability

Throughout this study, criteria established by various researchers to ensure validity and reliability in qualitative research (Creswell, 2013; Merriam, 2018; Miles & Huberman, 1994) were taken into account. A purposive sampling method was employed, and the rationale for participant selection, along with detailed descriptions of the participants, was provided. The data collection instrument, data collection procedures, timing, and data analysis methods were explicitly documented.

For inter-coder reliability, the researchers applied the formula proposed by Miles and Huberman (1994), ∆ = C ÷ (C + ∂:) × 100, calculating an agreement percentage of 92%. An expert review was conducted during data collection, data analysis, and the writing of the Findings, Discussion, and Conclusion Sections. Direct quotations were incorporated into the findings to enhance credibility. After the research report was completed, member checking was conducted to verify the accuracy of the data, and efforts were made to maintain objectivity throughout the research process.

## 3. Results

This study explores the perceptions of pre-service primary school teachers and pre-service elementary mathematics teachers regarding the ideal learning environment for mathematical activities through their drawings and related reflections. These perceptions were examined through their drawings and corresponding explanations.

The findings indicate that five key components (themes) emerge in the pre-service teachers’ perceptions of an ideal learning environment: purpose, instructional methods and techniques, student role, teacher role, and seating arrangement. The results suggest that these five components are dynamically interconnected, with the purpose component having a particularly significant influence on the other components.

### 3.1. Purpose Component

The pre-service teachers’ drawings and responses revealed various goals within the learning environment. Prominent goals included conveying mathematical knowledge, deepening understanding, promoting collaborative learning, and developing problem-solving skills. It was mainly observed that the purpose component significantly influences the other components. For instance, when the goal was identified as conveying mathematical knowledge, it was often associated with the lecture method, students taking on a passive listener role, and the teacher assuming the knowledge provider role.

The perceptions of teacher candidates regarding the purpose component of an ideal learning environment where mathematical activities are utilized are presented in Table 3.

A significant proportion of pre-service primary school teachers (54.17%) and pre-service elementary mathematics teachers (64.10%) aimed to concretize mathematical concepts. The drawings designed for this purpose included tools such as geometric shapes, abacuses, and number blocks, which facilitate students’ learning through tactile and manipulative experiences. The results obtained from the interviews revealed that pre-service teachers used concrete materials in their activity designs to make mathematics, an abstract subject, more tangible and to enhance its comprehensibility for primary and middle school students.

In addition to concretizing mathematical concepts, some pre-service teachers focused on deepening mathematical understanding. This objective was reported by 39.58% of pre-service primary school teachers, whereas the proportion was lower among pre-service elementary mathematics teachers. Notably, the emphasis on deepening understanding emerged more prominently in the interviews than in the drawings. In other words, while explaining their drawings, pre-service teachers justified their choice of objects by emphasizing that they enable students to understand a mathematical concept more comprehensively and relationally, as well as through question-and-answer interactions.

The role of the teacher in knowledge transmission also emerged as a key theme. In drawings where the teacher assumed a central role—writing on the board, providing explanations, and delivering information while students remained passive listeners and note-takers—the primary focus appeared to be the direct transmission of mathematical knowledge. Among the participants, 33.33% of pre-service primary school teachers and 17.95% of pre-service elementary mathematics teachers emphasized knowledge transmission within a learning environment that involved mathematical activities.

The integration of technology into the learning environment was another notable aspect of the drawings. While 33.33% of pre-service primary school teachers and 35.90% of pre-service elementary mathematics teachers incorporated technology, their representations predominantly featured digital tools such as interactive whiteboards, tablets, and computers. Additionally, drawings that depicted physical and pedagogical elements designed to enhance student interaction, collaborative learning, and social skill development—such as round tables, flexible seating arrangements, and games promoting equal participation—indicated a focus on fostering collaborative and social learning. The emphasis on this approach was observed in 10.42% of pre-service primary school teachers and 56.41% of pre-service elementary mathematics teachers.

**Figure 2:**
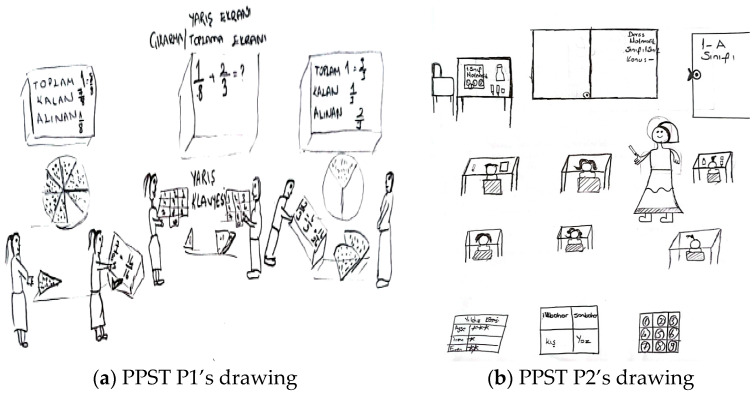
Drawings by pre-service primary school teachers in terms of the purpose component.

When explaining their drawings, pre-service teachers frequently mentioned ensuring that students learn through enjoyment, making lessons engaging, and increasing motivation, all of which suggest that they aimed to foster a positive attitude toward mathematics. This tendency was more pronounced in the interviews with pre-service primary school teachers. Additionally, although less frequently observed in both drawings and interviews, references were made to connecting mathematics to daily life, considering individual differences, enhancing mathematical communication skills, and interdisciplinary integration. For example, Figure 2a,b present the drawings of pre-service primary school teachers.

In relation to the drawing presented in Figure 2a, PPST P1 stated: “*Given that we live in a technological era, I designed a learning environment that leverages children’s interest in technology to support their concrete understanding of the topic ‘Fractions and Operations with Fractions’. In this environment, students first work with model pizzas divided into slices. When a student places the slices on a specially designed screen, the upper display shows the corresponding fraction representations under the headings ‘Total’, ‘Remaining’, and ‘Taken’. By performing operations such as expanding fractions, students earn points, making this activity—designed as an individual game—engaging and reinforcing their conceptual mastery. Additionally, students collaboratively combine pizza slices of different proportions at the center and complete the operations using keyboards. The student who completes the calculations the fastest wins the game. Through this competitive element, the learning process becomes more enjoyable and engaging*”. Figure 2a illustrates students learning operations with fractions. The students are playing a collaborative game with concrete materials in front of a computer screen at the center of the classroom. Although the teacher is absent, the pre-service teacher’s explanations suggest that they created the learning environment and provided guidance. Both the drawing and the pre-service teacher’s perspective indicate the purposes of integrating technology (e.g., using keyboards), concretizing mathematical concepts (e.g., using model pizzas to represent fractions), deepening mathematical understanding (e.g., enhancing mastery of the topic), and fostering a positive attitude toward mathematics (e.g., engaging students’ interests, making the learning process enjoyable through competition). In relation to the drawing presented in Figure 2b, PPST P2 provided the following explanation: “*In my opinion, children should be seated in this manner in a learning environment where mathematical activities take place. I depicted the children as first-grade students. There are various boards at the back of the classroom. Since they are newly learning numbers, I wrote the numbers on the boards. The teacher is delivering the lesson, and the students are working individually at their desks*”. It is possible to state that both the drawing and the pre-service teacher’s statements reflect a perception of a traditional learning environment in which the teacher is active and primarily aims to convey mathematical knowledge.

**Figure 3:**
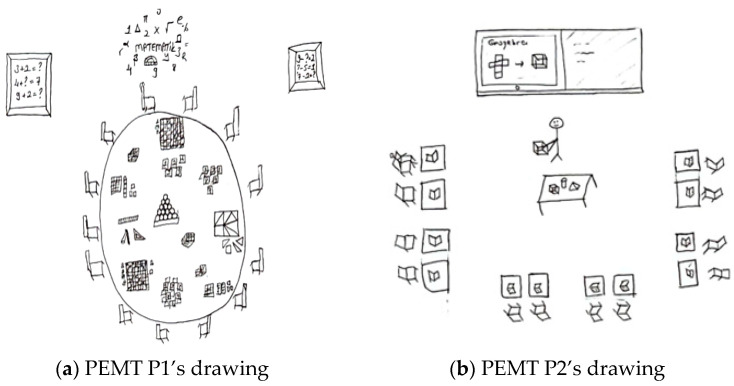
Drawings by pre-service elementary mathematics teachers in terms of the purpose component.

Figure 3a,b contain drawings by pre-service elementary mathematics teachers related to the purpose component.

In relation to the drawing presented in Figure 3a, a PEMT P1 stated the following: “*In my view, an ideal learning environment should be intelligence-enhancing, student-centered, and arranged in a face-to-face seating layout where students can see one another. The classroom I designed is specifically intended to create an enjoyable and engaging atmosphere for primary school students and those who experience difficulties in mathematics. In this setting, students will play games using chess, Rubik’s cubes, rulers, compasses, numbers, and tangrams. Additionally, they will explore mathematical concepts in a fun way by learning place values with hundred, ten, and unit blocks*”. Figure 3a shows a classroom designed in a circular arrangement, with concrete materials placed on the table but no visible students or teacher. The drawing and the pre-service teacher’s perspective suggest the goals of concretizing mathematical concepts (e.g., learning place values with hundred, ten, and unit blocks), considering individual differences (e.g., providing an enjoyable environment for students struggling with mathematics), supporting collaborative and social learning (e.g., students can see each other and play games together), and fostering a positive attitude toward mathematics (e.g., making learning fun). Regarding the drawing presented in Figure 3b, a PEMT P2 stated the following: “*I believe that in an ideal learning environment where mathematical activities are utilized, technology should be actively integrated. Classrooms should incorporate both whiteboards and interactive boards, and each student should have access to a computer. This would facilitate the understanding of real-life-related problems and be particularly beneficial for comprehending geometry through more effective activities. Additionally, concrete materials should be brought into the classroom. The teacher can use these materials to explain geometric objects and demonstrate their properties to students*”. In both the drawing and the pre-service teacher’s statements, it is evident that the teacher candidate aims to integrate technology, concretize mathematical concepts, convey mathematical knowledge, and relate it to daily life.

### 3.2. Teaching Methods and Techniques Component

Different teaching methods and techniques were prominently featured in the candidates’ drawings. The lecture method was one of the most frequently used techniques, while collaborative learning and problem-solving stood out. In some cases, it was noted that the methods and techniques chosen by the candidates were inconsistent with other learning environment components. For example, a teacher-centered lecture method was used despite the preference for a collaborative seating arrangement (e.g., U shape).

Pre-service teachers’ perceptions regarding the teaching methods and techniques for an ideal learning environment for mathematical activities are presented in Table 4.

As shown in Table 4, pre-service primary school teachers predominantly preferred direct instruction in the ideal learning environment for mathematical activities. The direct instruction method is used in traditional, teacher-centered learning environments, where the teacher transmits mathematical knowledge and students passively listen. Additionally, 22.92% of pre-service primary school teachers chose the question–answer technique, where the teacher asks students questions to assess learning and check understanding, with minimal student–teacher interaction, positioning the teacher in an evaluative role. Unlike pre-service primary school teachers, the question–answer technique was not reflected in the drawings of pre-service elementary mathematics teachers. Nine pre-service primary school teachers and three pre-service elementary mathematics teachers included educational games, physical movement games, competitive games, or gamified activities in their drawings, planning for active student participation.

In contrast to the views and drawings of pre-service primary school teachers, more than half of the pre-service elementary mathematics teachers considered collaborative learning to be a more ideal method in the learning environment for mathematical activities. In the drawings of teachers who preferred the collaborative learning method, the focus was on students working in small groups, interaction, and collaborative participation, with the teacher assuming a mentoring role. After collaborative learning, pre-service elementary mathematics teachers favored the lecture and demonstration methods. The demonstration method was chosen by four pre-service primary school teachers and seven pre-service elementary mathematics teachers. In the drawings and views using the demonstration method, the teacher demonstrates the activity practically, and students learn by replicating the same task, with the teacher serving as a model and the student being dependent on the teacher.

**Figure 4:**
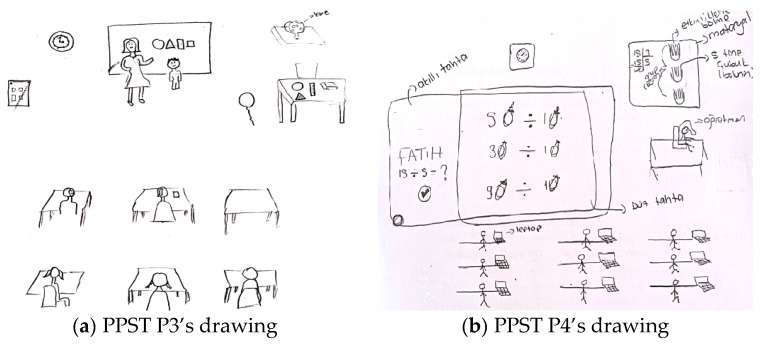
Drawings by pre-service primary school teachers in terms of the teaching methods and techniques component.

The problem-solving method was the least preferred technique overall. For example, Figure 4a,b present the drawings of pre-service primary school teachers.

In relation to the drawing presented in Figure 4a, PPST P3 stated the following: “*The teacher brings objects in the shape of triangles, circles, rectangles, and squares, and after showing these shapes to the children on the board and explaining their names, the teacher asks a student to go to the board and show triangular-shaped objects to the class. I drew a clock, a board, a pizza slice, a book, a balloon, a sphere, and a window for the class*”. In Figure 4a, the teacher explains geometric shapes on the board, such as triangles, circles, rectangles, and squares. A student is beside the teacher, being asked questions. There is a sphere-shaped material on one table, and objects like a pizza slice and a ruler are present on another. The students are seated individually, listening to the teacher, without communication or interaction. The drawing and the pre-service teacher’s comments suggest that the direct instruction method (e.g., after showing these shapes to the children on the board and explaining their names) and the question-and-answer technique (e.g., the teacher asks a student to go to the board and show triangular-shaped objects to the class) are being used. In relation to the drawing presented in Figure 4b, PPST P4 stated the following: “*I designed an activity like this. Every time a student answers a question on the smartboard, this symbol will light up. Students will also have laptops connected to the smartboard. This way, both time will be saved, and students’ technological literacy will improve. During the lesson, questions about shortcut division by 10 will be asked. Here, I replaced 0 with a balloon and placed chocolate under the balloon to motivate the students*”. It is possible to state that both the drawing and the pre-service teacher’s statements highlight the perception of a technology-supported classroom environment and that the pre-service teacher employs the question-and-answer technique.

**Figure 5:**
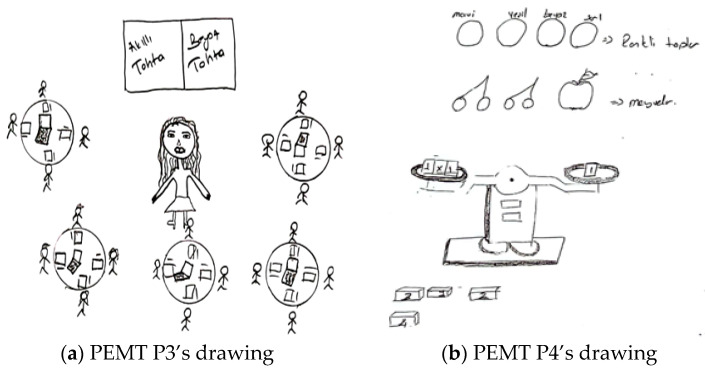
Drawings by pre-service elementary mathematics teachers in terms of the teaching methods and techniques component.

Figure 5a,b contain drawings by pre-service elementary mathematics teachers related to the teaching methods and techniques component.

In relation to the drawing presented in Figure 5a, PEMT P3 stated the following: “*I designed the classrooms for 20 students. There are 5 tables in the classroom, each accommodating 4 students. I chose a round table shape because I want students to engage in activities collaboratively in groups. The groups should be arranged heterogeneously. The teacher should act as a guiding mentor in this setup. Computers, paper, and pens are available on the tables for student use*”. In Figure 5a, the students are seated at round tables in a cluster arrangement, facing each other. Both a smartboard and a whiteboard are available in the classroom. Computers, paper, and pens are present on the students’ tables. The students are engaged in collaborative participation, with the teacher acting as a guide (e.g., the teacher should act as a guiding mentor), and technology is integrated into the learning environment. This pre-service teacher employs the collaborative learning method (e.g., students work collaboratively in groups). Regarding the drawing presented in Figure 5b, PEMT P4 stated the following: “*The activities may vary depending on the topic to be covered. The materials used in the activities may also differ accordingly. I designed a balance scale activity to teach the concept of equality. The teacher introduces balance to students using concrete materials. By placing objects of equal weight on both sides of the scale, the teacher models the concept. Then, the teacher calls the students one by one and asks them to balance the scale themselves. After explaining the concept of balance, the teacher will proceed to teaching equation solving*”. In Figure 5b, there are colored balls, fruits, and an image of a balance scale. However, there are no students or a teacher depicted in the classroom. Nevertheless, the pre-service teacher’s explanations indicate that the teacher employs the demonstration and guided practice technique by modeling the procedural steps.

### 3.3. Student Roles Component

The findings indicate that students’ roles in the learning environment vary widely. In some instances, students are depicted as passive listeners, while in others, they are described as active participants or collaborative group members. These roles are shaped in direct relation to the teaching methods and techniques employed.

The perceptions related to the student role component reflected in the pre-service teachers’ drawings and views regarding the ideal learning environment for mathematical activities are presented in Table 5.

As shown in Table 5, pre-service primary school teachers perceive the learning environment as one in which students predominantly participate as passive listeners, as reflected in their drawings and related comments. In drawings where the teacher conveys information through direct instruction, and the student assumes a passive role in receiving information, the student is depicted as a passive listener. In drawings where students show minimal participation in the learning process and answer questions, minimal interaction is observed. 22.92% of pre-service primary school teachers depicted students in the passive minimal interaction role. This role did not appear in the drawings or comments of pre-service elementary mathematics teachers.

In drawings where the teacher acts as a model and students imitate the teacher’s actions, demonstrating dependency on the teacher and guidance, the students assume a role of dependency on the teacher. In four of the pre-service primary school teachers’ drawings and seven of the pre-service elementary mathematics teachers’ drawings, this perception of student dependency on the teacher is predominant. Furthermore, 20.83% of pre-service primary school teachers depicted students in the active behavioral participation role. This percentage was lower among pre-service elementary mathematics teachers. In drawings where students actively participate physically in class activities, they are depicted in the active behavioral participation role.

In drawings showing problem-solving and discovery-based learning, where students are mentally engaged, they take on the role of active cognitive participation. The perception of students in the role of active cognitive participation did not appear among pre-service primary school teachers. However, it was present in three of the pre-service elementary mathematics teachers’ drawings.

In total, 56.41% of pre-service elementary mathematics teachers depicted students actively participating in mathematical activities, taking an active role within groups in a collaborative learning environment, where the collaborative teaching method is dominant.

**Figure 6:**
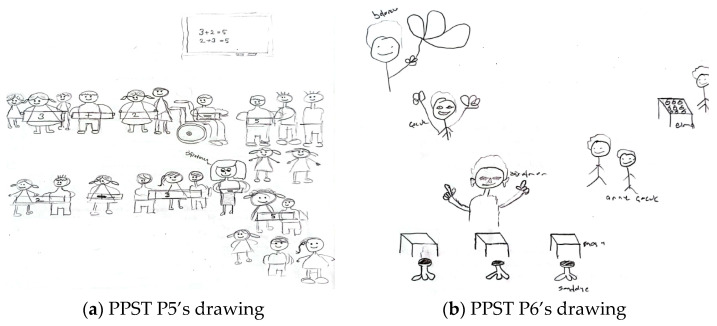
Drawings by pre-service primary school teachers in terms of the student role component.

In total, 25% of pre-service primary school teachers and 10.26% of pre-service elementary mathematics teachers perceived the role of students as one of active emotional participation in the ideal learning environment. This role is reflected in drawings and comments where students are emotionally engaged in learning, showing a positive attitude, emotional interest, engagement, enjoyment, and high motivation. In drawings where the learning environment aims to develop a positive attitude toward mathematics, students’ active emotional participation and the teacher’s role in providing emotional support are emphasized. For example, Figure 6a,b present the drawings of pre-service primary school teachers.

In relation to the drawing presented in Figure 6a, PPST P5 stated the following: “*The teacher is explaining the commutative property of addition, meaning that the result does not change even if the order of the addends is swapped. To ensure that students understand and retain this concept more easily, the teacher applies it practically with the students. In this way, the students learn while working collaboratively and having fun at the same time*”. In Figure 6a, the teacher aims to help students grasp the commutative property of addition. All the students are in the center of the class, engaging in the activity collaboratively. In this drawing and the related commentary, the pre-service teacher perceives the student role in the ideal learning environment as one of collaborative engagement (students are engaged in hands-on activities together, thus collaborating) and emotional engagement (they both have fun and learn). Regarding the drawing presented in Figure 6b, PPST P6 stated the following: “*The teacher should create an artificial environment in the classroom to explain the division operation. To help students transfer their learning to real life, a role-playing activity is conducted where parents, with a certain amount of money in their pockets, take their child to the market. Questions such as ‘How many apples can you buy if each costs 5 liras?’ are asked. In this way, real-life scenarios are brought into the classroom. In another role, I drew a balloon seller. The balloon seller states the unit price of a balloon, and the child states the amount of money they have. Then, the balloon seller can ask, ‘How many balloons can you buy with the money you have? …*” The pre-service teacher preferred the drama-based learning method. It is possible to state that both the pre-service teacher’s drawing and explanation reflect the perception of an ideal learning environment in which the student takes on an active behavioral engagement role.

**Figure 7:**
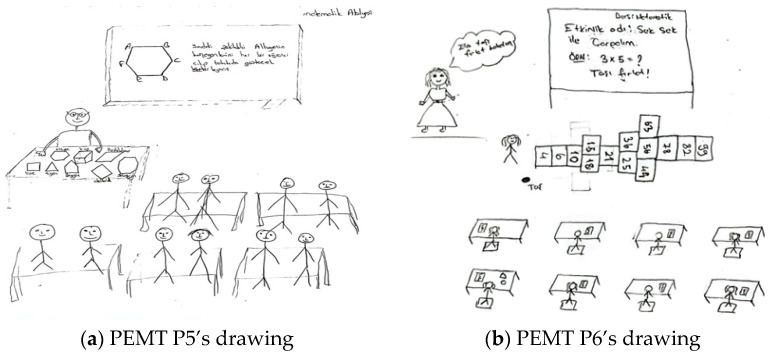
Drawings by pre-service elementary mathematics teachers in terms of the student role component.

Figure 7a,b contain drawings by pre-service elementary mathematics teachers related to the student role component.

In relation to the drawing presented in Figure 7a, PEMT P5 stated the following: “*The ideal math workshop classroom should have 10 students. First, the conditions such as lighting, sound, and temperature in this classroom should be within normal standards. The teacher should use materials to teach the topic more effectively. After introducing the materials (geometric shapes) to the students and explaining their features, the teacher should continue the lesson by using the discovery-based teaching method, encouraging active participation from the students*”. In Figure 7a, the teacher’s desk contains geometric objects such as cubes, triangles, and parallelograms, suggesting an intention to concretize mathematical concepts. The students are seated in a traditional row arrangement, with the teacher explaining the topic while the students listen. In this drawing, the pre-service teacher depicts the student as a passive listener and the teacher as the information provider. However, the pre-service teacher stated that the teacher first used direct instruction and then employed the discovery learning technique. In other words, the pre-service teacher also emphasized the role of students’ active cognitive engagement. Regarding the drawing presented in Figure 7b, PEMT P6 stated the following: “*In this activity, I used the traditional hopscotch game, which is well known in our culture, to develop students’ cognitive and psychomotor skills independently of technology. Since I aimed for a more active activity, I designed a game that moves away from individualism, where students interact closely and play together in a collaborative manner within the classroom*”. Since the drawing in Figure 7b depicts students engaging individually in the game physically, it is possible to state that the students take on an active behavioral engagement role. However, the pre-service teacher emphasized collaborative play in their statement. In other words, the pre-service teacher also highlighted the role of students’ active collaborative engagement.

### 3.4. Teacher Roles Component

The roles of teachers were explicitly illustrated through drawings and descriptions. Teachers, often defined as either “knowledge providers” or “guides”, occupy a central position in pre-service teachers’ perceptions of an ideal learning environment. However, the role of the teacher is directly related to the objectives of the learning environment and the methods employed.

Table 6 presents the components of the teacher’s role as reflected in pre-service teachers’ drawings and statements regarding an ideal learning environment in which mathematical activities take place.

As shown in Table 6, pre-service primary school teachers primarily perceived teachers as instructors–knowledge providers and mentors–guides in learning environments where mathematical activities take place. In drawings where the teacher assumes the knowledge provider role, the emphasis is primarily on the transmission of mathematical knowledge in a setting where students are mostly passive, and direct instruction is the preferred method. In contrast, in drawings where students were actively engaged in the learning process, with the teacher acting as a facilitator rather than an authoritative figure transmitting knowledge, the teacher assumed the guide role. In these drawings, pre-service teachers frequently incorporated collaborative learning and game-based teaching approaches. Furthermore, in depictions where teachers took on the guide role, they were often positioned among students rather than at the front of the classroom.

In total, 61.54% of pre-service elementary mathematics teachers perceived the teacher as a guide in an ideal learning environment. However, while 22.92% of pre-service primary school teachers represented teachers in the instructor–evaluator role, this perception was absent in the drawings and statements of pre-service elementary mathematics teachers. The evaluator role was typically illustrated in scenes where the question-and-answer technique was employed and students had minimal interaction.

Four pre-service primary school teachers and seven pre-service elementary mathematics teachers depicted the teacher in the model role, where students were dependent on the teacher and a demonstration-based teaching technique was used. The emotional support provider role was evident in the drawings and statements of both groups. In depictions where the primary goal of the learning environment was to foster a positive attitude toward mathematics, teachers were portrayed as providing emotional support. At the same time, students were shown as being actively engaged in emotional participation.

Additionally, three pre-service elementary mathematics teachers represented the teacher in the organizer role. In these drawings and statements, emphasis was placed on the teacher’s function of organizing the classroom environment and learning process, planning activities, distributing tasks, and refraining from direct intervention.

**Figure 8:**
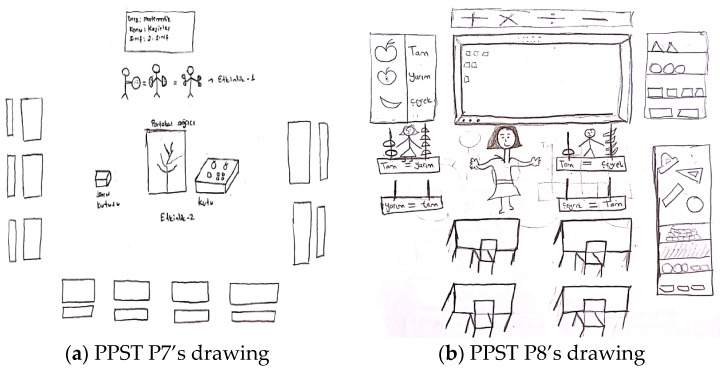
Drawings by pre-service primary school teachers in terms of the teacher role component.

For example, Figure 8a,b present the drawings of pre-service primary school teachers.

In relation to the drawing presented in Figure 8a, PPST P7 stated the following: “*The teacher designed two activities to explain the whole-half-quarter relationship in fractions. The classroom desks were arranged in a U-shape to ensure that students could easily see the activities being conducted. In Activity 1, the teacher introduced students to the relationship between whole, half, and quarter using pizza-shaped cutouts made of cardboard. Through these tangible materials, the teacher demonstrated that one whole is equivalent to two halves or four quarters. In Activity 2, the teacher presented a scenario in which an orange tree needed its oranges. Students were asked to solve the questions from a question box to help place the oranges back on the tree*”. In Figure 8a, the teacher is shown teaching fractions. There are three students in the classroom, each holding tangible materials to help them understand the whole–half–quarter relationship. In the center, there is a tree model and a question box. The teacher is not present in the classroom. The seating arrangement is depicted in a U-shape. The pre-service teacher aims to materialize mathematical concepts and relate them to real-life situations. Although the pre-service teacher draws the students engaged in the activity, their views emphasize direct instruction by the teacher (e.g., in Activity 1). In this context, the teacher assumes the knowledge provider role. At the same time, the pre-service teacher has designed an activity using the question-and-answer technique (e.g., in Activity 2). In this case, the teacher takes on the evaluator role. Regarding the drawing presented in Figure 8b, PPST P8 stated the following: “*While teaching the topic of fractions, I used a platform with two identical bars in the ideal classroom. This way, I explained the relationship between one whole and four quarters using concrete materials. Then, I invited students to the board and asked them to do the same. I also hung various posters in the classroom for whole, half, and quarter concepts. Additionally, I explained the topic by demonstrating with geometric shapes that can be combined and separated*”. In Figure 8b, it is observed that the teacher demonstrates the relationship between whole, half, and quarter using concrete objects for the students. Additionally, in their explanation of the drawing, the pre-service teacher states that the teacher illustrates this relationship in fractions by separating and combining various geometric objects. It is possible to state that the pre-service teacher, who employs the demonstration and guided practice technique, perceives the teacher as having a modeling role in an ideal learning environment.

**Figure 9:**
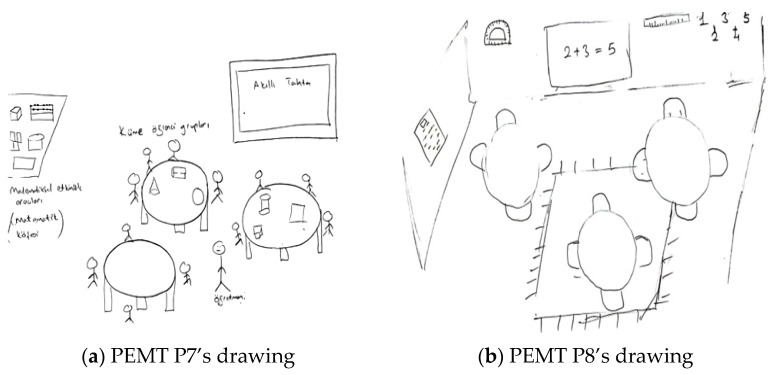
Drawings by pre-service elementary mathematics teachers in terms of the teacher role component.

Figure 9a,b contain drawings by pre-service elementary mathematics teachers related to the teacher role component.

In relation to the drawing presented in Figure 9a, PEMT P7 stated the following: “*Student groups collaborate either individually or in teams, following a cooperative learning approach. The interactive whiteboard plays a significant role in enriching the content with digital materials related to the activity’s construction, objectives, and other relevant aspects. Each learning environment should include a mathematics corner, which contains mathematical activities or objects appropriate to the class level, along with the materials used for the activities. The teacher should assume the role of a guide, offering support without directly intervening in the students’ work*”. In Figure 9a, students are seated at round tables in a cluster arrangement. Various objects are placed on the tables. The teacher is among the students but does not intervene directly. The classroom features a corner with concrete materials like abacuses and cubes and an interactive whiteboard. It is clear that the pre-service teacher aims to materialize mathematical concepts and integrate technology into the learning process. Based on the pre-service teacher’s views (e.g., students work individually or in groups following a collaborative learning approach.) and the seating arrangement, it is evident that a collaborative learning approach has been adopted, with students playing an active role in collaborative participation. The pre-service teacher’s choice to depict the teacher as not intervening directly and stating their views suggests that the teacher is taking on the organizer role. Regarding the drawing presented in Figure 9b, PEMT P8 stated the following: “*The number of students in the classroom should be small because this ensures that the education provided is efficient for each student. This way, the teacher can listen to each student’s questions and opinions. The classroom should be spacious and should not have traditional desks, as these desks hinder a flexible learning environment. Instead, there could be separate tables to allow students to engage in group activities. Depending on the activity, there could also be a carpet where students can feel comfortable. The teacher can assist students during group activities. This space was designed as an activity room. The walls feature mathematics-related materials and pictures. Additionally, portraits of renowned mathematicians can be displayed, which may increase students’ interest in mathematics*”. In Figure 9b, there are no teachers or students depicted in the classroom. However, it is possible to state that the pre-service teacher has drawn a flexible learning environment that allows students to engage in collaborative group work. It is evident that the pre-service teacher envisions the teacher in a guiding role while students work in groups. Additionally, the pre-service teacher’s perspective reflects the perception of the teacher as an emotional support provider, aiming to foster a positive attitude toward mathematics.

### 3.5. Seating Arrangement Component

In this study, the seating arrangement used in the learning environment varied. The drawings predominantly reflected traditional rows representing a typical classroom setting, U-shaped arrangements supporting collaborative learning, or group seating arrangements. However, instances were observed where the seating arrangement did not align with other components. For example, while a U-shaped arrangement, which is expected to promote communication and interaction, was preferred, a teacher-centered instructional method was still employed.

The components of seating arrangement, as reflected in the drawings and perspectives of pre-service teachers regarding an ideal learning environment for mathematical activities, is presented in Table 7.

As shown in Table 7, pre-service primary school teachers perceive the flexible arrangement as the ideal seating arrangement in an optimal learning environment. However, this arrangement was not widely preferred by pre-service elementary mathematics teachers. Pre-service teachers who envisioned a flexible seating arrangement depicted a classroom without a fixed seating plan, where students could freely choose their seats based on their needs, the activity, or personal preference.

Among pre-service primary school teachers, 25% represented the seating arrangement as U-shaped, while 25.64% of pre-service elementary mathematics teachers depicted the same arrangement. Along with the U-shaped arrangement, pre-service elementary mathematics teachers most frequently preferred the grouped arrangement. The circular arrangement, which is generally intended for group work or discussion settings, was the least frequently drawn seating arrangement by pre-service teachers overall.

**Figure 10:**
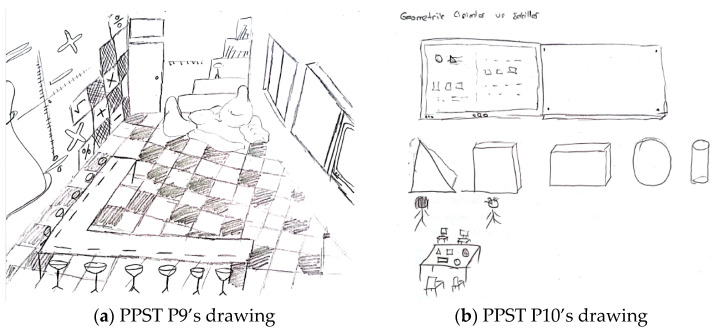
Drawings by pre-service primary school teachers in terms of the seating arrangement component.

For example, Figure 10a,b present the drawings of pre-service primary school teachers.

In relation to the drawing presented in Figure 10a, PPST P9 stated the following: “*The cabinets on the walls should both evoke mathematics and provide children with personal spaces. The classroom should have both a smartboard and a traditional blackboard. In my opinion, mathematics is more enjoyable to teach on a blackboard. I envisioned a classroom that includes both a traditional learning environment and a comfortable space for activities, where mathematical and logic-based games can be conducted*”. In Figure 10a, the pre-service primary school teacher preferred a flexible seating arrangement. Instead of traditional desks, the classroom features stools and cushions in one corner. The floor and walls of the classroom are depicted differently from those in conventional classrooms. In their statement, the pre-service teacher emphasized using a smartboard, the importance of students engaging in activities in a comfortable environment, and the opportunity to play various games. Regarding the drawing presented in Figure 10b, PPST P10 stated the following: “*To teach geometric solids, I bring three-dimensional shapes that I have made at home and allow students to examine them. I explain properties such as vertices, edges, and surfaces using these shapes. Then, I ask students to draw, cut, and create the same shapes from paper or cardboard based on what I have taught. I also show examples on the smartboard*”. The pre-service teacher’s explanations indicate that the teacher employs both direct instruction and the demonstration and guided practice method. While the pre-service teacher’s perspective reflects the perception of students as passive listeners who are dependent on the teacher, the drawing incorporates a group-based classroom arrangement.

**Figure 11:**
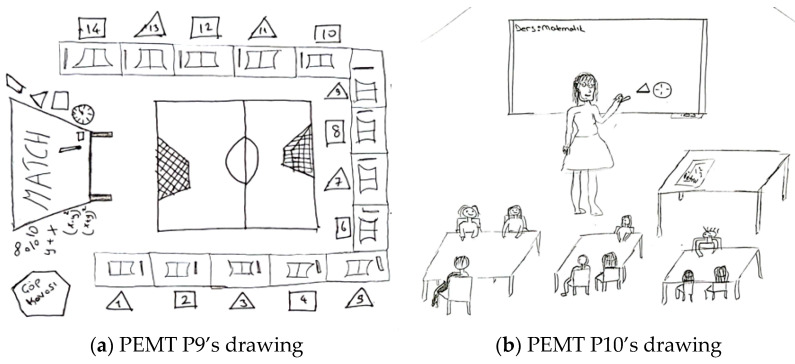
Drawings by pre-service elementary mathematics teachers in terms of the seating arrangement component.

Figure 11a,b contain drawings by pre-service elementary mathematics teachers related to the seating arrangement component.

In relation to the drawing presented in Figure 11a, PEMT P9 stated the following: “In my opinion, the seating arrangement in an ideal learning environment should be like this. Since students are seated side by side, they do not disturb each other. They can support one another, enabling them to learn and apply the collaborative learning style. The floor should feature visuals that capture students’ interest, as they should be able to use them for role-playing while playing games. The classroom walls should include visuals and materials that enhance students’ interest and curiosity in mathematics. Additionally, students’ chairs should be designed in geometric shapes such as squares and triangles”. In Figure 11a, the pre-service elementary mathematics teacher opted for a U-shaped seating arrangement. The students’ seats were drawn to resemble various geometric shapes. The pre-service teacher highlighted that this seating arrangement allows students to work collaboratively without disturbing one another. The classroom walls contain mathematical symbols, and the floor features football-themed drawings, which the pre-service teacher believed would capture students’ interests. Regarding the drawing presented in Figure 11b, PEMT P10 stated the following: “The classroom environment is designed to encourage students to form small groups and work together toward a common goal to complete a given activity or task. After explaining the topic, the teacher can assign a task and ask students to complete it collaboratively in groups”. Figure 11b represents a mathematics classroom environment that adopts a collaborative learning approach. Students are divided into small groups and seated around tables. The teacher first explains the lesson on the board after which students engage in activities together. It is possible to state that the pre-service teacher prefers a group-based arrangement in an ideal learning environment.

While the relationships between different dimensions are more apparent in the pre-service teachers’ drawings, it is impossible to generalize about the learning environment based solely on the seating arrangement. For instance, in a classroom where learning through play or a collaborative learning approach is employed, it is evident that cooperative and social learning are prioritized, with the teacher generally acting as a guide and the student taking on an active behavioral or collaborative participation role. However, some drawings revealed that the purpose of the seating arrangement did not always align with its intended use. For example, in classrooms with a traditional row arrangement or an individual seating arrangement, some pre-service teachers emphasized discovery learning and students’ cognitive engagement in their statements.

## 4. Discussion and Conclusions

Pre-service teachers’ perceptions of ideal learning environments for mathematical activities are shaped around five key components: purpose, instructional methods and techniques, student roles, teacher roles, and seating arrangements. It was found that a dynamic relationship exists among these components, with the purpose component exerting a particularly strong influence on the others.

Pre-service primary school teachers placed a greater emphasis on objectives such as concretizing mathematical concepts and fostering a positive attitude toward mathematics. This may stem from their perception that mathematics instruction at the primary school level should be made comprehensible and engaging for students. Their choice of instructional methods aligns with this tendency; their preference for techniques supported by tangible materials, such as direct instruction and teaching through games, reflects their efforts to design accessible and easily understandable student activities.

In contrast, pre-service elementary mathematics teachers focused on promoting collaborative and social learning while also emphasizing the concretization of mathematical concepts. Their preference for collaborative learning and guided practice methods highlights their pedagogical priorities at the middle school level, particularly in fostering students’ problem-solving skills and engaging them in cognitively demanding activities. The widespread adoption of collaborative learning among pre-service elementary mathematics teachers suggests an emphasis on peer interaction and higher-order thinking skills.

These differing approaches can be directly linked to the requirements of instructional levels and the pedagogical priorities of pre-service teachers. Considering the student level, pre-service primary school teachers tend to concretize mathematical concepts and foster a positive perception of mathematics to make them more accessible to their target student groups. In contrast, pre-service elementary mathematics teachers emphasize engaging in mathematical activities within a social environment, which is reflected in their activity designs. Although pre-service teachers attempt to create student-centered learning environments in their activities, teacher-centered approaches remain dominant in both groups. This predominance may stem from their instructional experiences, where teacher-centered pedagogies prevail. Additionally, factors such as large class sizes in undergraduate education, the physical classroom environment being designed to support traditional approaches, and the predominance of teacher-centered pedagogies in most courses further reinforce this perception. The theoretical knowledge of student-centered approaches is often delivered to pre-service teachers through teacher-centered pedagogies, and their limited practical experience with constructivist environments may have hindered their ability to conceptualize the components of student-centered learning environments. For instance, McMinn et al. (2021) noted that positively perceived learning environments enhance pre-service teachers’ self-efficacy in mathematics instruction. However, these environments can sometimes be associated with more traditional beliefs. This finding brings the balance between teacher-centered and student-centered approaches into discussion. Elen et al. (2007) suggested that the relationship between teacher-centered and student-centered learning environments can be examined from different perspectives. They argued that dynamics such as balance, independence, or renegotiation influence the quality of learning environments. However, their study emphasized that teacher-centered and student-centered environments can have mutually reinforcing characteristics, advocating for a focus on developing strong learning environments rather than merely transitioning between the two approaches.

The tendency of pre-service teachers to design more student-centered learning environments is evident in the study by Eroğlu and Arslan (2024). Their research revealed that pre-service teachers’ ideal mathematics learning environment drawings predominantly featured student-centered designs. However, Toprak et al. (2017) found that these approaches are not always adequately reflected in practice, highlighting the implementation gap between teacher-centered approaches and student-centered objectives. Xenofontos and Hizli Alkan (2022) suggested that such implementation deficiencies may also be linked to a lack of professional awareness. These researchers emphasized the importance of pre-service teachers gaining more professional experience outside of formal learning environments to enhance their mathematical awareness, proposing initiatives such as Mathematics Fairs to foster this awareness.

The impact of classroom social climate on learning environments was examined by Kuzle (2023). Their study demonstrated that the social climate in geometry classrooms directly influences students’ perceptions and interactions, underscoring the necessity of more interactive learning environments. The design of interactive learning environments not only enriches students’ learning experiences but also reshapes pre-service teachers’ pedagogical priorities. In the present study, pre-service primary school teachers’ emphasis on concretization and fostering positive attitudes suggests that they conceptualize the learning environment within a more traditional framework. Meanwhile, pre-service elementary mathematics teachers’ focus on promoting collaborative and social learning reflects an effort to encourage complex thinking skills within a student-centered approach.

The perception of pre-service primary school teachers, who generally position students as passive listeners and emotional participants, reflects the need for a focus on sensory and individual learning at the elementary school level. In contrast, pre-service elementary mathematics teachers’ emphasis on collaborative participation indicates that they view the development of student interactions and problem-solving skills as a fundamental pedagogical element. These findings align with the literature, emphasizing student-centered learning approaches in contemporary learning environments. By assuming social and cognitive roles that shape their educational experiences, students are encouraged to engage more actively, enhancing the quality of learning outcomes (Damşa & de Lange, 2019; Yu, 2022). Furthermore, viewing students as co-authors of the curriculum contributes to ensuring equality and democracy in learning environments (Vogt, 2022). In this regard, the approaches of pre-service elementary mathematics teachers, which encourage collaborative learning, represent a constructivist understanding aimed at developing student interaction and joint problem-solving skills.

Pre-service primary school teachers tend to position the teacher as a provider of knowledge and a guide, whereas pre-service elementary mathematics teachers prioritize the roles of guide and model. This suggests that pre-service primary school teachers strive to balance traditional teaching methods with student-centered guidance, while pre-service elementary mathematics teachers focus on supporting independent thinking and problem-solving processes. The literature highlights that teachers collaborating with students in personalized learning environments to support their individual needs is critical in creating an effective learning experience (Bishop et al., 2020; Putri et al., 2019). Moreover, the transition to interactive and participatory teaching methods requires teachers to design knowledge transfer as a bidirectional process in line with constructivist approaches (Kumar, 2014). In this context, the emphasis placed by pre-service elementary mathematics teachers on their roles as guides and models indicates that they are closer to a student-centered teaching approach.

Seating arrangement preferences provide important insights into how teacher candidates’ methods and techniques relate to their perceptions of the learning environment. The preference of pre-service primary school teachers for free and U-shaped seating arrangements can be explained by their emphasis on individual and emotional learning. On the other hand, pre-service elementary mathematics teachers’ preference for group-based and U-shaped seating arrangements is consistent with collaborative learning approaches. The literature suggests that seating arrangements significantly affect student participation and learning dynamics. For instance, U-shaped arrangements encourage active participation, while group-based arrangements enhance social interaction and collaboration among students (Hayashi et al., 2023; Hilal, 2014). Additionally, circular and flexible arrangements are noted to strengthen the sense of community and belonging, positively impacting learning experiences (Falout, 2014). The preference for group seating by pre-service elementary mathematics teachers highlights their emphasis on collaborative learning, while the preference for more traditional arrangements by pre-service primary school teachers signals differences in their pedagogical priorities.

Another significant finding of this study is the notable difference between the perceptions of pre-service primary school teachers and pre-service elementary mathematics teachers regarding ideal learning environments. Pre-service primary school teachers generally focused on concrete and easily understandable examples, preferring activities facilitating individual or group work. In contrast, pre-service elementary mathematics teachers conceptualized the concept of activity at a more abstract level, independent of a specific context, within the learning environment.

The fact that teaching and learning are social and cultural activities (Brion, 2022) suggests that these differing approaches to learning environment components stem from the professional training, depth of subject knowledge, and pedagogical priorities of teacher candidates. The interpretation of learning environments is influenced by the positions, institutional affiliations, and cultural backgrounds of different educational stakeholders, which affect their development and implementation of educational practices (Kovač et al., 2017). The differences observed in the components can be attributed to the varying levels at which pre-service primary school teachers and pre-service elementary mathematics teachers engage with students and the differentiation of curriculum content.

In conclusion, this study reveals that the components shaping pre-service teachers’ perceptions of ideal learning environments interact dynamically, with a notable connection to the goal-oriented dimension. The findings underscore the importance of restructuring educational programs to ensure that teaching methods and techniques align more effectively with the established goals. Specifically, it is recommended that more hands-on training and feedback processes be integrated into the programs to enhance teacher candidates’ skills in learning environment design. This study concludes that teacher education programs should be designed to support the alignment of candidates’ teaching methods and techniques with their goals more consistently.

## 5. Limitations and Future Research

In this study, the data are limited to teacher candidates’ drawings and opinions regarding learning environments involving mathematical activities. The messages conveyed in the drawings may not represent the complex reality of teaching and learning situations. The drawings may be the product of teacher candidates’ perceptions of a particular phenomenon or event or their expected perception.

This study is limited to qualitative data obtained from pre-service primary school teachers and pre-service elementary mathematics teachers from the Faculty of Education of a single university. As this study was conducted with a qualitative design, the results may be shaped by the researchers’ perspectives and influenced by their experiences. It is a well-established fact that teaching and learning environments affect perceptions. The findings of this study suggest that the majority of teacher candidates do not have a constructivist perception of learning environments. One of the factors contributing to this situation may be the teacher candidates’ experiences. As we have previously stated, the constructivist learning approach is addressed theoretically in undergraduate courses for pre-service teachers; however, the courses are conducted using traditional learning approaches. Therefore, future research could be conducted with teacher candidates from education faculties in different universities.

In order to reflect the data as accurately as possible, teacher candidates were told that they could provide written explanations alongside their drawings. Additionally, after completing the drawings, interviews were conducted with the teacher candidates for verbal explanations of their drawings. This approach aimed to minimize our interpretations. However, since the researchers did not review the drawings prior to conducting these interviews, some unclear points were identified during the coding process. For instance, we created codes based on the positions and sizes of objects in the drawings and the classroom seating arrangements. Interpretation may come into play here. Future studies could address this issue by conducting multiple interviews.

In this study, the pre-service primary school teachers are all enrolled in the 3rd year of their program, while the pre-service elementary mathematics teachers include both 3rd- and 4th-year students. The reason for selecting 3rd-grade pre-service primary school teachers is that they take the “Mathematics Teaching” course in their 3rd year, as per the national curriculum in Türkiye. By the time the research data were collected, they had learned teaching and learning strategies to be used in mathematics instruction, the scope, objectives, and characteristics of the primary school mathematics curriculum, major learning theories and their relationships with mathematics learning, and the use of information technologies in mathematics. We believed we would obtain better data since they engaged in mathematical activities in this course. In the elementary mathematics teaching program, however, teaching courses (Teaching Numbers, Teaching Algebra, Teaching Geometry and Measurement, and Teaching Probability and Statistics) are offered in both the third and fourth years, which is why we selected pre-service elementary mathematics teachers from both years. However, there were more volunteer candidates from the 4th year. This may be because, in the 4th year, teacher candidates take the “Teaching Practice” course and have internships in schools, which may have influenced their perceptions. Future studies could work with groups that share more similar characteristics.

One particular finding in this study was that pre-service primary school teachers generally drew a typical day of a single mathematical activity rather than depicting a classroom environment with mathematical activities. To address this, future research could ask participants to create multiple drawings. Moreover, some teacher candidates expressed concerns about their drawings’ poor quality and their lack of drawing skills, which may have prevented them from fully reflecting their perceptions in their drawings. Future studies involving adults could provide opportunities for electronic drawing, which may help overcome this issue.

## Figures and Tables

**Table 1:** Demographic characteristics of the participants.

Group	Characteristic	Distribution	Percentage	Frequency
Pre-Service Elementary Mathematics Teachers	Gender	Male	25.6%	10
		Female	74.4%	29
	Age	24+	20.5%	8
		23	46.2%	18
		22	23.1%	9
		21	10.2%	4
	Class	3rd year	10.2%	4
		4th year	89.8%	35
	Course or Project	Participated	12.8%	5
		Not Participated	87.2%	34
Pre-Service Primary School Teachers	Gender	Male	29.2%	14
		Female	70.8%	34
	Age	24+	25.1%	12
		23	22.9%	11
		22	29.1%	14
		21−	22.9%	11
	Class	3rd year	100%	48
		4th year	0%	0
	Course or Project	Participated	10.4%	5
		Not Participated	89.6%	43

**Table 2:** Operational definitions of classroom characteristics in student drawings.

Themes	Sub-Themes	Operational Definitions
**Purpose**	Concrete Representation of Mathematical Concepts	Enhancing the understanding of abstract concepts through visual, manipulative, or technological tools.
	Deepening Mathematical Understanding	Ensuring that students develop a deep rather than superficial comprehension of mathematical concepts.
	Integrating Technology	Integrating digital resources to enhance the teaching and learning of mathematics.
	Supporting Collaborative and Social Learning	Promoting student collaboration to facilitate meaningful learning experiences.
	Developing a Positive Attitude Toward Mathematics	Enhancing students’ confidence and motivation by making mathematics enjoyable.
	Conveying Mathematical Knowledge	Conveying mathematical ideas clearly, accurately, and effectively to students.
	Relating to Daily Life	Establishing connections between mathematical concepts and real-world contexts.
	Considering Individual Differences	Adapting teaching methods to accommodate diverse learning styles and needs.
	Enhancing Mathematical Communication Skills	Enhancing students’ ability to express mathematical thinking both orally and in writing.
	Interdisciplinary Connections	Providing meaningful learning opportunities by connecting mathematics with other subject areas.
**Teaching Methods and Techniques**	Direct Instruction	A teaching method in which the teacher systematically and explicitly conveys information to students.
	Question-and-Answer Method	A strategy that structures learning by prompting students with questions to encourage thinking and active participation.
	Game-Based Learning	A method that actively engages students in the learning process through educational games.
	Demonstration Method	A technique in which the teacher models a process step by step, allowing students to replicate it.
	Discovery Learning	A method in which students construct knowledge independently through guided exploration and inquiry.
	Collaborative Learning	A strategy that promotes learning through small-group work, where students cooperate toward a shared goal.
	Problem-Solving Method	An approach that fosters critical thinking and decision-making skills by engaging students in solving real-world or hypothetical problems.
	Learning Through Drama	A method in which students experience learning through activities such as role-playing, dramatization, and simulations.
**Student Role**	*Active Student*	
	Behavioral Engagement	Contributes to the learning process by participating in activities, completing tasks, and taking active roles in the classroom.
	Cognitive Engagement	Focuses on cognitive processes such as deep thinking, analysis, and problem-solving.
	Emotional Engagement	Connects to the learning process with positive emotions, demonstrating curiosity, interest, and motivation.
	Collaborative Engagement	Actively participates in group work and cooperates effectively with peers.
	*Passive Student*	
	Passive Listening	Merely listens to the teacher’s explanations without making an effort to understand or providing feedback.
	Minimal Interaction	Shows minimal participation in the lesson, rarely responding to questions or engaging in activities.
	Teacher Dependence	Fully relies on the teacher’s guidance throughout the learning process and does not take independent initiative.
**Teacher Role**	*Instructor*	
	Knowledge Provider (Resource Person)	Contributes to the learning process by presenting necessary information in an accurate, clear, and systematic manner.
	Evaluator	Monitors students’ learning progress, assesses their development, and provides constructive feedback.
	Role Model	Serves as an example through behaviors and attitudes, helping students develop positive learning habits.
	*Mentor*	
	Guide (Advisor)	Provides guidance throughout the learning process, offering strategies to help students achieve their goals.
	Organizer (Planner)	Plans the educational process, organizes activities, and effectively structures the learning environment.
	*Emotional Support Provider*	Responds sensitively to students’ emotional needs, fostering a sense of trust, motivation, and belonging.
**Seating Arrangement**	Flexible Seating Arrangement	An adaptive seating arrangement that allows students to move freely and interact, accommodating different learning needs.
	U-Shaped Arrangement	A setup where students sit in a semicircle, facilitating face-to-face interaction with the teacher and encouraging group discussions.
	Individual Seating Arrangement	A structure in which each student sits alone, typically in rows, promoting individual work and concentration.
	Traditional (Row) Arrangement	A setup where students sit in rows, making it suitable for teacher-centered instruction.
	Group (Cluster) Arrangement	A configuration where students sit in small groups, fostering collaborative learning and teamwork.
	Circle Arrangement	A seating arrangement in which students sit in a circular formation, promoting equal participation and open discussions.

**Table 3:** Purpose component in the learning environment.

Purpose	Pre-Service Primary School Teacher (f = 48)	Pre-Service Elementary Mathematics Teacher (f = 39)
Concretizing Mathematical Concepts	54.17% (26)	64.10% (25)
Deepening Mathematical Understanding	39.58% (19)	2.56% (1)
Integrating Technology	33.33% (16)	35.90% (14)
Supporting Collaborative and Social Learning	10.42% (5)	56.41% (22)
Developing Positive Attitudes Toward Mathematics	25% (12)	10.26% (4)
Conveying Mathematical Knowledge	33.33% (16)	17.95% (7)
Relating to Daily Life	12.5% (6)	7.69% (3)
Considering Individual Differences	2.08% (1)	10.26% (4)
Developing Mathematical Communication Skills	-	5.13% (2)
Interdisciplinary Connection	2.08% (1)	-

**Table 4:** Teaching methods and techniques component in the learning environment.

Teaching Methods and Techniques	Pre-Service Primary School Teacher (f = 48)	Pre-Service Elementary Mathematics Teacher (f = 39)
Direct Instruction	33.33% (16)	17.95% (7)
Question–Answer	22.92% (11)	-
Game-Based Learning	18.75% (9)	7.69% (3)
Demonstration Method	8.33% (4)	17.95% (7)
Discovery Learning	-	5.13% (2)
Collaborative Learning	10.42% (5)	56.41% (22)
Problem-Solving Method	-	2.56% (1)
Learning Through Drama	8.33% (4)	-

**Table 5:** Student role component in the learning environment.

Student Role		Pre-Service Primary School Teachers (f = 48)	Pre-Service Elementary Mathematics Teachers (f = 39)
Passive Student	Passive Listening	33.33% (16)	17.94% (7)
	Minimal Interaction	22.92% (11)	-
	Dependency on Teacher	8.33% (4)	17.95% (7)
Active Student	Behavioral Participation	20.83% (10)	15.38% (6)
	Cognitive Participation	-	7.69% (3)
	Emotional Participation	25% (12)	10.26% (4)
	Collaborative Participation	10.42% (5)	56.41% (22)

**Table 6:** Teacher role component in the learning environment.

Teacher Role		Pre-Service Primary School Teachers (f = 48)	Pre-Service Elementary Mathematics Teachers (f = 39)
Instructor	Knowledge Provider	33.33% (16)	17.95% (7)
	Evaluator	22.92% (11)	-
	Model	8.33% (4)	17.95% (7)
Mentor	Guide	33.33% (16)	61.54% (24)
	Organizer	-	7.69% (3)
Emotional Support Provider		25% (12)	10.26% (4)

**Table 7:** Seating arrangement component in the learning environment.

Seating Arrangement	Pre-Service Primary School Teachers (f = 48)	Pre-Service Elementary Mathematics Teachers (f = 39)
Flexible (Adaptive) Arrangement	27.08% (13)	10.26% (4)
U-Shaped Arrangement	25% (12)	25.64% (10)
Individual Arrangement	16.67% (8)	7.69% (3)
Traditional (Row) Arrangement	10.42% (5)	10.26% (4)
Grouped (Cluster) Arrangement	4.17% (2)	25.64% (10)
Circular (Round) Arrangement	2.08% (1)	12.82% (5)

## Data Availability

The data presented in this study are available on request from the corresponding author due to ethical considerations.

## Abbreviations

The following abbreviations are used in this manuscript:

PPST	Pre-service primary school teacher
PEMT	Pre-service elementary mathematics teacher
